# A Rapid Method to Regenerate Piezoelectric Microcantilever Sensors (PEMS)

**DOI:** 10.3390/s110505520

**Published:** 2011-05-20

**Authors:** LiNa Loo, Wei Wu, Wan Y. Shih, Wei-Heng Shih, Hossein Borghaei, Kambiz Pourrezaei, Gregory P. Adams

**Affiliations:** 1 Developmental Therapeutics Program, Fox Chase Cancer Center, 333 Cottman Ave., Philadelphia, PA 19111, USA; E-Mails: lina.loo@fccc.edu (L.L.); hossein.borghaei@fccc.edu (H.B.); 2 Department of Materials Science and Engineering, Drexel University, Philadelphia, PA 19104, USA; E-Mails: wuweifly@gmail.com (W.W.); shihwy@drexel.edu (W.Y.S.); shihwh@drexel.edu (W.-H.S.); pourrezaei@gmail.com (K.P.)

**Keywords:** piezoelectric microcantilever, biosensor, regeneration, antibody, antigen, biomarker

## Abstract

Piezoelectric microcantilever sensors (PEMS) can be sensitive tools for the detection of proteins and cells in biological fluids. However, currently available PEMS can only be used a single time or must be completely stripped and refunctionalized prior to subsequent uses. Here we report the successful use of an alternative regeneration protocol employing high salt concentrations to remove the target, leaving the functional probe immobilized on the microcantilever surface. Our model system employed the extracellular domain (ECD) of recombinant human Epidermal Growth Factor Receptor (EGFR) as the probe and anti-human EGFR polyclonal antibodies as the target. We report that high concentrations of MgCl_2_ dissociated polyclonal antibodies specifically bound to EGFR ECD immobilized on the sensor surface without affecting its bioactivity. This simple regeneration protocol both minimized the time required to re-conjugate the probe and preserved the density of probe immobilized on PEMS surface, yielding identical biosensor sensitivity over a series of assays.

## 1. Introduction

Label-free microcantilever biosensors have recently been the focus of significant developmental efforts for use in biomedical applications including disease screening [1], blood glucose monitoring [2], and detection of cancer biomarkers such as prostate specific antigen [3]. Piezoelectric microcantilever sensors (PEMS) consisting of a piezoelectric layer, lead magnesium niobate–lead titanate [4,5], (PbMg_1/3_Nb_2/3_O_3_)_0.63_–(PbTiO_3_)_0.37_(PMN-PT) bonded to a nonpiezoelectric layer such as tin have shown particular promise for label-free detection in real time. When a PEMS probe (e.g., an antibody) binds to its target (e.g., an antigen) in a biological fluid a shift occurs in the PEMS resonance frequency, which can be measured in real time [6,7]. PEMS are particularly attractive for these applications as they undergo electric self-excitation and self-sensing and are capable of withstanding liquid damping, thereby allowing highly sensitive measurements to be performed in biological fluids. They can be functionalized by immobilizing receptors or target biomolecules to their surface to facilitate the highly specific binding of target molecules such as antigens or even intact cells. The resonance frequency shifts can then be detected by electrical means for further analysis. Using this approach, we have detected extremely low concentrations of human HER2, a breast cancer associated biomarker, in serum using PEMS functionalized with anti-HER2 single chain Fv (scFv) engineered antibody-based molecules [6].

An ideal biosensor would embody basic features such as selectivity, specificity and reproducibility. Specificity and selectivity are readily obtained by functionalizing the surface of the PEMS with probes (e.g., anti-HER2 antibodies) that selectively capture the desired analytes (e.g., HER2). However, the reuse of functionalized PEMS sensors in a reproducible manner has not yet been reported. Typically harsh techniques, such as glycine-HCl mixture at pH 2.5 [4,11] or a “Piranha Solution” composed of sulfuric acid and hydrogen peroxide [8–10], have been employed to strip both the analyte and probe from the PEMS surface, often damaging or removing the insulation layer in the process. These procedures require the PEMS to be completely refunctionalized via the addition of entirely new insulation layers and biomolecule probes. As a result, the sensitivity of the probe is often altered [12,13]. Additional drawbacks to these techniques are that they are extremely time consuming in nature and they use significant quantities of often valuable and limited resources.

Interactions between antibodies and their cognate antigens involve a variety of factors: electrostatic, hydrogen bonds, Van der Waals and hydrophobic forces. These forces can be weakening/disrupted by using high salt and/or low pH buffer solutions. When employed properly, this process is reversible with little or no damage to the antibody or antigen. This principle has been applied in a wide variety of immunological studies including affinity-based target antigen-capture and release assays [14,15]. These approaches are commonly used to regenerate the surface plasmon resonance (Biacore) chips or Quartz Crystal Microbalance (QCM) [16,17]. However, to the best of our knowledge, this approach has not been previously employed with piezoelectric sensors. Our goal in the current study was to examine the ability of high salt concentrations to dissociate the target-probe complex, removing the analyte but leaving intact probe immobilized on PEMS surface. Our proof-of-concept model system employed the extracellular domain (ECD) of recombinant human Epidermal Growth Factor Receptor (EGFR) as the probe and goat anti-human EGFR polyclonal antibodies as the targets, but the procedures can be employed for all antibody-antigen interactions. After utilizing the PEMS biosensor to detect a target, the PEMS/EGFR ECD/anti-EGFR polyclonal antibody complex was immersed in MgCl_2_ to dissociate the probe/target complex. The target was stripped from the PEMS leaving the intact functional probe (EGFR ECD) on the surface for the next measurement. This straightforward regeneration protocol both minimized the time required to re-conjugate the probe and preserved the density of probe immobilized on PEMS surface, allowing identical sensitivity over a series of assays.

## 2. Experimental Section

### 2.1. PEMS Preparation for Assay

PEMS were constructed by bonding an 8 μm thick lead magnesium niobate-lead titanate (PMN-PT) freestanding film piezoelectric layer to a gold/copper nonpiezoelectric layer as previously described [5]. The PMN-PT/Cu was cut to the cantilever shape, with typical dimensions of 560 μm in length and 720 μm in width, with a wire saw (Princeton Scientific Precision, Princeton, NJ) after embedding in wax. Wires were attached to the top and bottom electrodes using conductive glue (XCE 3104XL Emerson and Cuming Company, Billerice, MA, USA), followed by using glue to attach the PMN-PT/Cu strips to a glass substrate. To insulate PEMS with mercaptopropyltrimethoxysilane (MPS), the PEMS was first soaked in a piranha solution, which contains two parts of 98% sulfuric acid (Fisher, Fair Lawn, NJ) with one part of 30% hydrogen peroxide (FisherBiotech, Fair Lawn, NJ, USA) at 20 °C for 1 min. The PEMS was submerged in 0.1 mM MPS (Sigma Aldrich) diluted in ethanol for 30 min before drying the PEMS in a vacuum-oven (Model 1400E, VWR International) at 762 mm Hg overnight. Next, the PEMS was submerged in a 1% (volume) of MPS diluted in ethanol (titrated to a pH 4.5 with acetic acid) for 36 h with the solution being changed every 12 h. The PEMS was then dried in a vacuum-oven (Model 1400E, VWR International) overnight at 762 mm Hg.

### 2.2. Immobilization of Extracellular Domain (ECD) of Recombinant Human Epidermal Growth Factor Receptor (EGFR) on PEMS

Recombinant human EGFR ECD was expressed from stably transfected HEK293 cells and purified as we have previously described [18]. 1 μM EGFR ECD was conjugated to 80 uM Sulfosuccinimidyl 4-N-maleimidomethyl cyclohexane-1-carboxylate (sulfo-SMCC) (Pierce) in 1 mL 1xPBS (Gibco) mixed with 5 mM EDTA, pH 7.4, for 30 min at room temperature. Excess sulfo-SMCC was then removed by filtration in a Microcon-10k (Millipore). The PEMS were then immersed in sulfo-SMCC-linked EGFR ECD solution for 30 min. Subsequently, PEMS functionalized with EGFR ECD were immersed in 3% BSA for 30 min to block the PEMS surface.

### 2.3. Detection of Anti-Human EGFR Polyclonal Antibody

**Figure 1.**
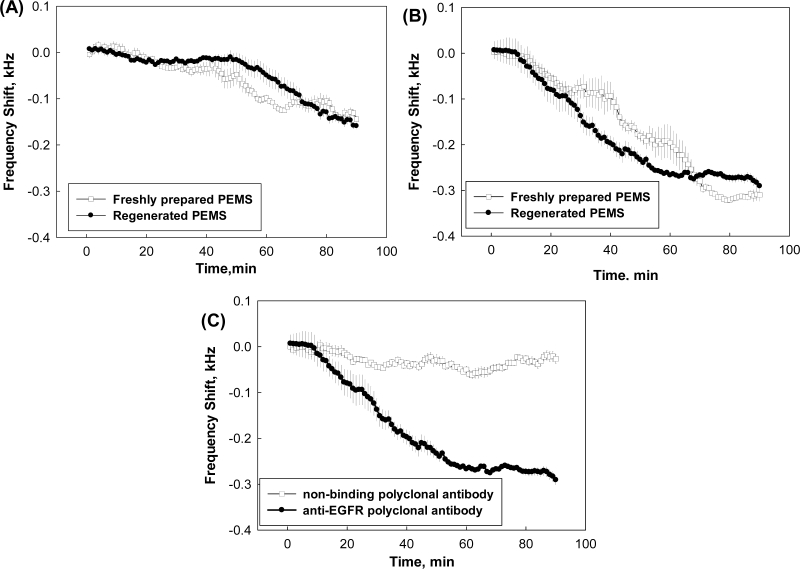
Comparison of frequency shifts observed when **(A)** 1 ng/mL and **(B)** 4 ng/mL of PAb_peptide_ were administered to a freshly prepared EGFR ECD conjugated PEMS (empty squares) and a regenerated PEMS (filled squares). For both concentrations, the regenerated PEMS yields a frequency shift that was similar to the freshly prepared PEMS with the recovery rate described in text. **(C)** Comparison of frequency shifts resulting from the binding of 4 ng/mL of specific PAb_peptide_ anti-EGFR polyclonal antibodies (filled squares) and 4 ng/mL of non-binding, non-reactive polyclonal antibodies (empty squares) to a regenerated EGFR ECD conjugated PEMS. The injection of non-binding antibody yielded a minimal frequency shift, suggesting that non-specific binding was not a major factor.

After BSA treatment, PEMS functionalized with EGFR ECD were incubated in a 3.5 mL custom-built flow cell containing 3 mL PBS-EDTA. A peristaltic pump (model 77120-62, Cole-Parmer’s Master Flex, Vernon Hills, IL), was then used to flow PBS-EDTA at the low rate of 0.7 mL/min in a perpendicular direction relative to the PEMS surface. Once a stable baseline was obtained for a period of at least 20 min, anti-human EGFR polyclonal antibodies diluted in PBS were injected into the system and the frequency shift was measured for 90 min with an electrical impedance analyzer (Agilent 4294A, Agilent). Two formulations of anti-human EGFR polyclonal antibodies were used in this study; anti-EGFR (1005) polyclonal antibodies, PAb_peptide_, raised against a peptide located at the C-terminus of human EGFR (Santa Cruz Biotechnology Inc) and polyclonal antibodies directed against recombinant full length human EGFR, PAb_full-length_, (Abnova). An anti-rabbit polyclonal antibody (Santa Cruz Biotechnology, sc-2030) was used as a negative control to study the specificity of the PEMS. This anti-rabbit polyclonal antibody, referred as the non-binding polyclonal antibody in Figure 1(C), recognized rabbit polyclonal antibody and not the probe (EGFR ECD) immobilized on PEMS.

### 2.4. Piranha Solution Stripping and Refunctionalization

Stripping of PEMS with piranha solution was performed as previously described [5]. Briefly, after each detection run, the PEMS were immersed in a 1:100 diluted piranha solution (two parts of 98% sulfuric acid and one part of 30% hydrogen peroxide) for one minute, followed by immersion in water and ethanol for 30 s each. The PEMS were then incubated in 40 mM solution of MPS in ethanol overnight, before drying in a vacuum-oven. The PEMS were then insulated and refunctionalized as described above for new sensors.

### 2.5. Rapid PEMS Regeneration by High Salt Solution

After detection runs, PEMS were immersed in 2 M MgCl_2_ for 30 s, followed by 1.5 M Tris, pH 8.8 for an additional 30 s. The regenerated PEMS were incubated in 3% BSA for 30 min before being employed for a subsequent detection run.

### 2.6. Data Analysis

The longitudinal extension mode of PEMS vibration in the range of 200–1,000 kHz was used for data analysis as previously described [5]. Longitudinal mode was selected as liquid damping has the least effect on this mode. The relative frequency shift (Df/f) was calculated based on Equation (1) as below:

(1) $$\text{Df}/\text{f}=\left({\text{F}}_{\text{baseline}}-{\text{F}}_{\text{sample}}\right)/{\text{F}}_{\text{baseline}}$$

where F_baseline_ is the average 20 points of the baseline (before sample injection) and F_sample_ is calculated by averaging the last 20 points of the detection period. To compare the recovery of a regenerated PEMS-functionalized with EGFR ECD, an equation as below was used:

(2) Recovery (%)=(Df/f of a regenerated PEMS÷Df/f of a freshly prepared PEMS)×100 (%)

The significance of the differences in analyte detection between PEMS (freshly prepared, stripped and refunctionalized or regenerated) were determined using a two-tailed student’s t-test (GraphPad). P values < 0.05 were considered to indicate the presence of statistically significant differences.

## 3. Results and Discussion

### 3.1. Comparison of Freshly Prepared PEMS and Regenerated PEMS

Initial studies were performed examining the regeneration of EGFR ECD conjugated PEMS following their use in the detection of polyclonal antibodies, PAb_peptide_, directed against a single peptide sequence on EGFR ECD protein. As the antibodies used in this experiment were focused on a small region of the ECD, the quantity of antibodies capable of binding would be expected to be limited, potentially leading to a more straightforward regeneration process. A typical spectrum (frequency shift *vs.* time) observed for 1 ng/mL of PAb_peptide_ binding to a freshly prepared EGFR ECD-conjugated PEMS is shown in Figure 1(A) (empty squares). The same PEMS was then regenerated using MgCl_2_ as described in the methods section and equilibrated with PBS buffer to reach a stable baseline. The regenerated EGFR ECD-conjugated PEMS was then reassayed with PAb_peptide_ at 1 ng/mL (filled squares), yielding a frequency shift that was very similar to that observed with the freshly prepared PEMS (empty squares) (Figure 1(A)). The same PEMS was then cleaned with piranha solution, refunctionalized with new insulation and EGFR ECD and employed in the detection of a higher concentration (4 ng/mL) of PAb_peptide_. The resulting frequency shift is presented as empty squares in Figure 1(B). The PEMS was regenerated with MgCl_2_ and employed to detect the binding of 4 ng/mL of PAb_peptide_ (filled squares), again yielding a frequency shift that was similar to that seen with the freshly prepared PEMS. Each experiment was repeated three times and the average binding to the regenerated PEMS was highly comparable to that seen with the original freshly prepared or refunctionalized PEMS (101.80 ± 10.82% and 101.67 ± 7.50% at PAb_peptide_ concentrations of 1 ng/mL and 4 ng/mL, respectively). In summary, assays performed with both concentrations of PAb_peptide_ revealed that the regenerated EGFR ECD-conjugated PEMS exhibited no loss in ability to bind polyclonal anti-EGFR antibodies.

To eliminate the possibility that the frequency shifts observed in these assays were due to non-specific interactions, regenerated EGFR ECD conjugated PEMS were assayed with 4 ng/mL of non-binding polyclonal antibody (anti-rabbit polyclonal antibody) that was incapable of binding specifically to the EGFR ECD. The non-binding antibody yielded a minimal frequency shift (13.3% of the value observed with the specific PAb_peptide_) at the conclusion of the 90-minute measurement period (Figure 1(C)). This suggested that the binding of PAb_peptide_ to EGFR ECD immobilized on the PEMS surface was specific.

The ability of the regeneration procedure to remove a more complex mixture of polyclonal antibodies was examined next. For these studies, we employed polyclonal antibodies generated against the entire EGFR ECD. As the targeted sequence on the protein was significantly larger than that used in the studies described above, the mixture of antibodies likely contained a broader range of affinities and could potentially target multiple epitopes on each EGFR ECD molecule simultaneously. A range of different dilutions of PAb_full-length_ were assayed on regenerated PEMS-functionalized with EGFR ECD and compared to a freshly prepared PEMS-functionalized with EGFR ECD. As shown in Table 1, the observed Df/f values at the conclusion of the 90 min measurement period were not significantly different for the freshly prepared and regenerated PEMS, indicating that the regenerated PEMS were capable of binding the same quantity of polyclonal antibody. This demonstrated that PEMS regeneration with MgCl_2_ was capable of stripping a complex mixture of antibodies from the immobilized antigen yet gentle enough to leave the antigen probe intact.

Table 1 compares the full-length anti-EGFR polyclonal antibody (PAb_full-length_) detection by PEMS prepared by stripping with piranha solution and refunctionalizing with new insulation and EGFR ECD (freshly prepared) and PEMS treated with MgCl_2_ leaving the EGFR ECD intact on the sensor surface (Regenerated). The experiments were performed in triplicate and the Df/f were reported as average ±standard error. P values were >0.05 suggesting that the measurements between the freshly prepared PEMS and regenerated PEMS were in accordance.

### 3.2. Determination of the Reusability of MgCl_2_ Regenerated PEMS

An important requirement for assays employed in the development and validation of biosensors is the availability to reproducibly reuse the sensors without significant changes in sensitivity. This is particularly critical when small variations in the dimensions of manually prepared sensors can lead to disparate results. To address this, EGFR ECD conjugated PEMS were sequentially employed in a series of detection and regeneration cycles. In each cycle, a stable baseline was obtained, 2 ng/mL of PAb_peptide_ was applied to the sensor and the change in frequency was measured 90 min later. The PEMS was then regenerated as described above and the process was repeated. As shown in Table 2, we observed that the regeneration procedure was gentle enough to remove the bound polyclonal antibodies while still maintaining the quality of the EGFR ECD. The minimal frequency changes suggested that probe remained intact on the cantilever surface for at least three cycles (four consecutive measurements), after which the change in frequency was significantly altered. In our experience, PEMS can be stripped with piranha, reinsulated, refunctionalized and reused for at least five cycles without damaging the PEMS itself. Therefore, we feel that it is likely that the loss of PEMS’s ability to recover after three cycles of regeneration is due to one of two possibilities; (1) damage to the insulation layer which has historically been replaced entirely in the refunctionalization procedure after stripping with piranha solution or (2) damage to the probe itself which could result from its long exposure to room temperature during the repetitive measurements (>6 h).

Table 2 compares the recovery rate of regeneration cycle for the detection of 2 ng/mL anti-EGFR peptide polyclonal antibody. P values were calculated to compare the statistically difference between the indicated regeneration cycle and the previous regeneration cycle.

## 4. Conclusions

We have described a rapid and reliable regeneration procedure for the gentle dissociation of target proteins (e.g., antibody) from probes immobilized (e.g., antigen) on the surface of PEMS. This regeneration protocol allows PEMS-based sensors to be used sequentially with minimal loss of sensitivity. Further experiments are needed to validate the versatility of this regeneration protocol on PEMS for other antigen-antibody detections. However, we believe that the method can be widely extended to biosensors that rely on probe immobilization including receptor-ligand, antibody-antigen and enzyme-substrate interactions. As PEMS technology matures, reproducibility will likely be achieved through the use of standardized production of arrayed sensors that include internal controls, negating the need for regeneration. However, in the meantime, the availability of reliable regeneration procedures will be critical for the evaluation of new PEMS. In conclusion, the regeneration protocol described here gently removes target proteins from probes on the PEMS surface and facilitates the repetitive use of these biosensors.

## Figures and Tables

**Table 1.** Comparison between freshly prepared PEMS and regenerated PEMS.

Dilutions of PAb_full-length_	Df/f (×10^−4^)		P value
	Freshly prepared PEMS	Regenerated PEMS	
10^−6^	7.54 ± 1.29	6.05 ± 1.15	0.437
10^−9^	4.55 ± 0.79	5.13 ± 0.45	0.558
10^−11^	2.04 ± 0.38	1.50 ± 0.14	0.253
10^−12^	0.56 ± 0.08	0.56 ± 0.03	0.957

**Table 2.** Recovery rate of regenerated PEMS.

Regeneration cycle	Recovery (%)	P value
1	100	0.9336
2	95.90 ± 2.37	0.1587
3	93.93 ± 4.33	0.7102
4	87.76 ± 10. 91	0.6269
5	4.984 ± 2.879	0.0018
